# Insights into LATE from postmortem imaging

**DOI:** 10.1002/alz.083407

**Published:** 2025-01-09

**Authors:** Paul A. Yushkevich, Amanda E Denning, Lisa M Levorse, Ranjit Ittyerah, Niyousha Sadeghpour, John L. Robinson, Sanaz Arezoumandan, Sadhana Ravikumar, Daniel T Ohm, Theresa Schuck, David J Irwin, Eddie B Lee, Alejandra Bahena, Winifred Trotman, Sandhitsu R. Das, David A Wolk, Laura E.M. Wisse, Robin de Flores, Ricardo Insausti, Pulkit Khandelwal, Chinmayee Athalye

**Affiliations:** ^1^ Penn Alzheimer’s Disease Research Center, University of Pennsylvania, Philadelphia, PA USA; ^2^ University of Pennsylvania, Philadelphia, PA USA; ^3^ Perelman School of Medicine at the University of Pennsylvania, Philadelphia, PA USA; ^4^ Department of Neurology, Perelman School of Medicine, University of Pennsylvania, Philadelphia, PA USA; ^5^ Department of Diagnostic Radiology, Clinical Sciences, Lund University, Lund Sweden; ^6^ Normandie Univ, UNICAEN, INSERM, U1237, PhIND "Physiopathology and Imaging of Neurological Disorders", NeuroPresage Team, GIP Cyceron, Caen France; ^7^ University of Castilla‐La Mancha, Albacete Spain

## Abstract

**Background:**

Limbic‐predominant age‐related TDP‐43 encephalopathy (LATE) is a neurodegenerative disease that is often comorbid with Alzheimer's disease (AD) and for which there are no reliable specific chemical or PET biomarkers available. Recent progress in disease‐modifying treatments for AD elevates the need for reliable in vivo detection of LATE and other comorbid neurodegenerative diseases. The promise of postmortem and antemortem MRI studies in LATE is that they will lead to the discovery of patterns of neurodegeneration associated with TDP‐43 pathology that could be reliably detected in vivo and used as a biomarker of LATE.

**Method:**

Several groups, including ours, have imaged brain tissue (e.g., whole hemisphere, or intact temporal lobe) from autopsies conducted in older adults with and without cognitive impairment. Specimens were imaged using high‐field, high‐resolution magnetic resonance imaging (MRI) and quantitative maps of cortical thickness were derived. Statistical associations between these structural measures and either computationally derived quantitative or expert‐assigned semi‐quantitative markers of TDP‐43 and tau pathology are computed as statistical parametric maps and statistically thresholded to identify clusters of significant associations. Alternatively, such associations can be derived from antemortem imaging studies, where MRI is performed in living individuals and linked to postmortem pathology measures.

**Result:**

Both TDP‐43 and tau pathology are linked to cortical thinning in the medial temporal lobe, as revealed by postmortem and antemortem studies. Patterns of thinning associated with each pathology are distinct yet overlapping, and sensitive to cohort selection, choice of pathology measure, and other factors.

**Conclusion:**

Reliable in vivo detection of TDP‐43 based on in vivo MRI signatures is likely possible but requires effort from the community to pool scarce ex vivo imaging and pathology datasets, standardization of quantitative pathology and MRI measures, and extensive validation.

